# A new species of *Xorides* Latreille (Hymenoptera, Ichneumonidae, Xoridinae) parasitizing *Pterolophia alternata* (Coleoptera, Cerambycidae) in *Robinia pseudoacaci*a

**DOI:** 10.3897/zookeys.246.3853

**Published:** 2012-11-29

**Authors:** Mao-Ling Sheng, Rui-Xing Zhao, Shu-Ping Sun

**Affiliations:** 1 General Station of Forest Pest Management, State Forestry Administration, 58 Huanghe North Street, Shenyang 110034, P. R. China; 2 Liaoning Forestry Pest Control and Quarantine Bureau, Shenyang, Liaoning, 110804, P.R. China

**Keywords:** *Xorides*, new species, key, parasitoid wasp, idiobiont, *Pterolophia alternata*, Cerambycidae, host plant, China

## Abstract

A new species is described, *Xorides benxicus* Sheng, **sp. n.**, reared from the cerambycid twig-boring pest of *Robinia pseudoacacia* Linnaeus, *Pterolophia alternata* Gressitt, 1938, in Benxi County, Liaoning Province, China. A key is given to the species similar to *Xorides benxicus* Sheng, namely *Xorides asiasius* Sheng & Hilszczański, 2009, *Xorides cinnabarius* Sheng & Hilszczański, 2009 and *Xorides sapporensis* (Uchida, 1928).

## Introduction

*Xorides* Latreille, 1809, belonging to the subfamily Xoridinae of Ichneumonidae (Hymenoptera), comprises 159 described species (Hilszczański and Plewa 2011, Yu et al. 2012). 43 species have been reported from China (Liu and Sheng 1998, Sheng et al. 1998, 1999, 2004, 2006, 2008, 2009, 2010, Zong and Sheng 2009). The hosts are larvae of wood-boring Coleoptera, mainly Cerambycidae and Buprestidae (Clément 1938, Hilszczański et al. 2005, Sheng et al. 2002, 2010, Yu et al. 1997). The status of the genus was defined by Townes (1969) and Wahl (1997).

In this article a new species of *Xorides* is described. The species was reared in Benxi County, Liaoning Province, at the southern border of the Eastern Palearctic part of China, as a parasitiod of *Pterolophia alternata* Gressitt, 1938 (Coleoptera: Cerambycidae), which bores twigs of *Robinia pseudoacacia* Linnaeus and is considered a pest.

The type locality is a forest composed of mixed deciduous angiosperms and evergreen conifers, mainly including *Robinia pseudoacacia*, *Castanea* spp., *Quercus* spp., *Larix* sp., *Rosa multiflora* var. *cathayensis* Rehd. & Wils., *Rubus* sp. and *Pinus tabulaeformis* Carr.

## Materials and methods

Rearing parasitoids. Twigs of naturally heavily infested *Rubus pseudoacacia* trees were brought to the laboratory and maintained in a large nylon cage at room temperature. Water was sprayed over the trunks and twigs twice a week and emerged insects collected daily.

Rearing parasitoid larvae and pupae. Parasitoid larvae and cocoons were collected from galleries of wood-borers in infested twigs of *Rubus pseudoacacia* and stored individually in glass tubes with a piece of filter paper dipped in distilled water to maintain moisture and plugged tightly with absorbent cotton wool.

The host was identified by Professor Wen-Kai Wang, Changjiang University, Hubei Province, China.

Images of whole insects were taken using a CANON Power Shot A650 IS. Other images were taken using a Cool SNAP 3CCD attached to a Zeiss Discovery V8 Stereomicroscope and captured with QCapture Pro version 5.1.

The morphological terminology is mostly that of Gauld (1997). Wing vein nomenclature is based on Ross (1936) and the terminology on Mason (1986, 1990).

Type specimens and hosts are deposited in Insect Museum, General Station of Forest Pest Management, State Forestry Administration, P. R. China.

### Xorides

Latreille, 1809

http://species-id.net/wiki/Xorides

Xorides Latreille, 1809. Genera Crustaceorum et Insectorum secundum ordinem naturalem in familias disposita iconibus exemplisque plurimis explicata, 4:4. Type species: *Ichneumon indicatorius* Latreille.

#### Diagnosis.

Apex of mandible chisel-shaped, unidentate. Subapical part of female flagella elbowed or bent, on the outer profile of the elbow or bend several peg-like bristles. Epomia present, usually strong, dorsally turning forward and sharply projecting. Front tibia usually thickened. Second tergum with an oblique basal groove on basolateral corner. Apical part of ovipositor cylindric or slightly depressed, the lower valve with several ridges.

### Xorides benxicus

Sheng sp. n.

urn:lsid:zoobank.org:act:F9C857C8-B7D4-459C-A3F5-16B77FA18D26

http://species-id.net/wiki/Xorides_benxicus

**Figures 1–4.**
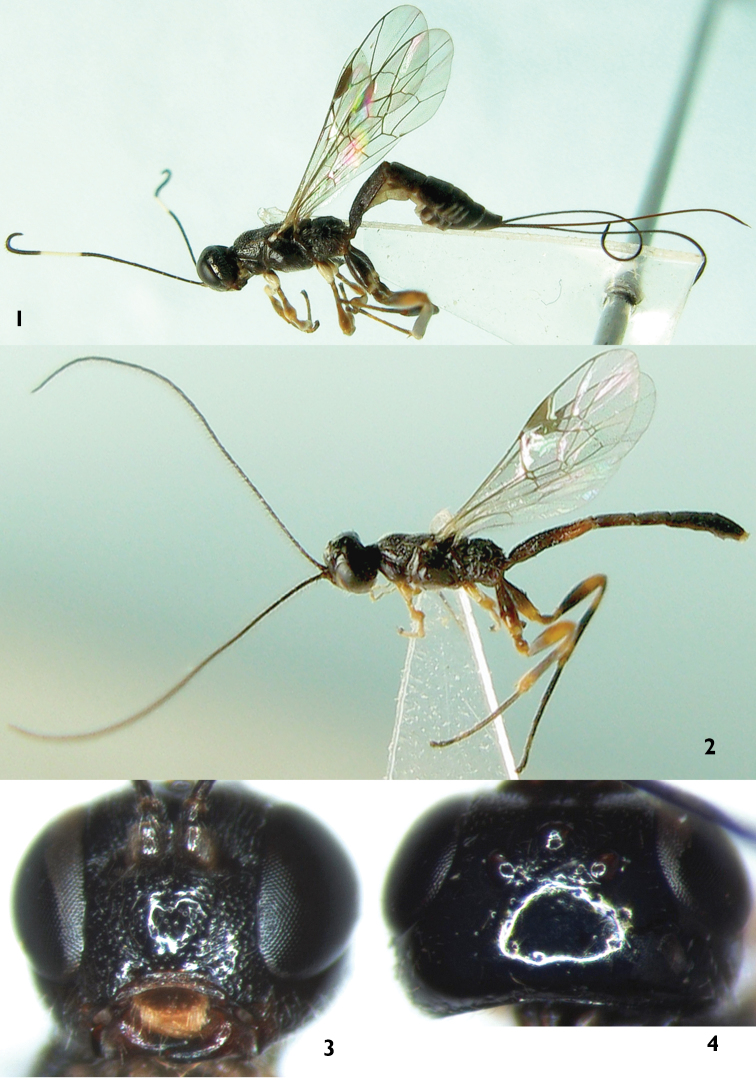
*Xorides benxicus* Sheng, sp. n. **1, 3–4** Holotype female **2** Paratype male **1,2** Body, lateral view **3** Head, anterior view **4** Head, dorsal view.

Figures 1 [Fig F2] 12

#### Etymology.

The name of the new species is based on the type locality.

#### Types.

*Holotype*, Female, CHINA: Benxi County, Liaoning Province, 19 June 2012, leg. Mao-Ling Sheng. Paratypes: 3 females and 2 males, same data as holotype, except 18 to 19 June 2012.

#### Diagnosis.

**Figures 5–9. F2:**
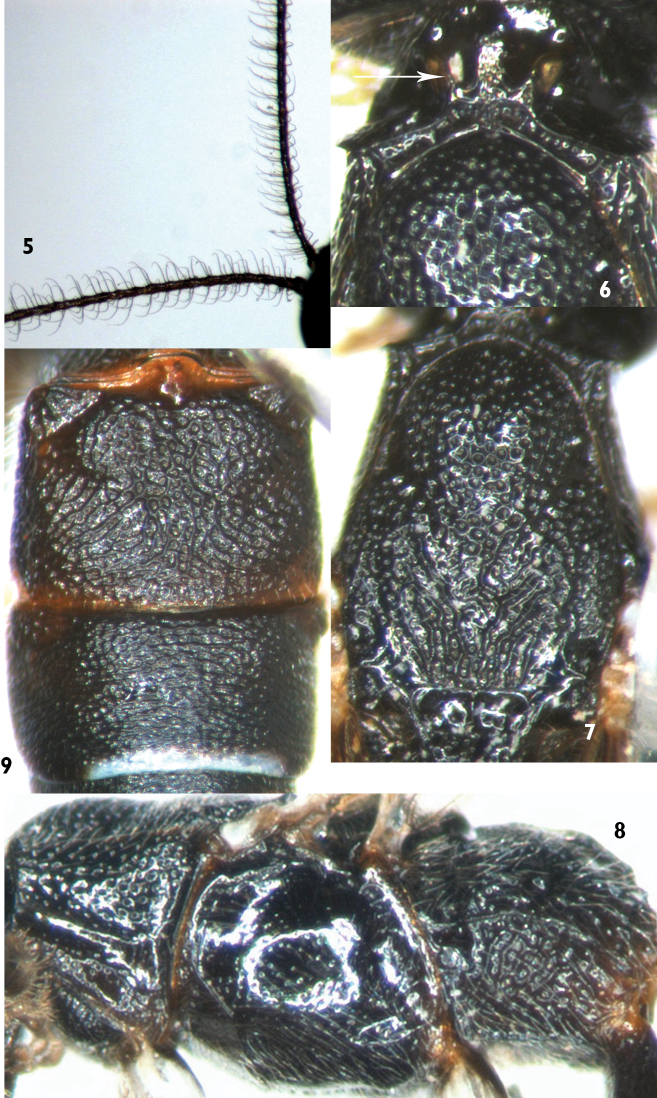
*Xorides benxicus* Sheng, sp. n. **5** Paratype male, basal portion of antenna **6–9** Holotype female **6** Pronotum and anterior portion of mesoscutum, dorsal view **7** Mesoscutum **8** Mesosoma, lateral view **9** Terga 2 & 3.

*Xorides benxicus* can be distinguished from the similar species of *Xorides*, possessing subapical terga with white apical spots in females, by the combination of the characters: head and mesosoma entirely black; face strongly convex centrally; apical part of lateral longitudinal carinae of area basalis combined; posterior part of second tergum with irregular longitudinal wrinkles; hind margins of terga 3 to 7 white, lateral parts of the white portions broken; last tergum with a smooth median longitudinal groove. Ovipositor sheath approximately 2.5 times as long as hind tibia. Flagella of male (Figure 5) slightly compressed, apex of each flagellomere swollen, lateral and ventral-lateral profiles with erect long setae, setae approximately 3.5 times as long as width of flagellomere and curved apically.

#### Description.

**Female.** Body length 5.5 to 7.5 mm. Fore wing length 4.3 to 5.5 mm. Ovipositor sheath length 4.2 to 5.5 mm.

**Head**. Face (Figure 3) approximately 1.8 times as wide as long, strongly convex, with uneven, fine punctures; median portion shining, sparsely punctate; lower-lateral portion with indistinct oblique wrinkles; upper portion with median longitudinal groove; upper margin with strong median projection towards frons. Clypeal suture distinct. Clypeus with sub-basal transverse ridge; below ridge strongly inclined, weakly concave, with fine coriaceous texture. Mandible with fine median longitudinal groove; basal portion with fine longitudinal wrinkles; tooth shining. Subocular sulcus distinct. Malar space 0.6 to 0.7 times as long as basal width of mandible. Inner part of subocular sulcus shining with very sparse punctures, outer part with distinct oblique wrinkles and fine punctures. Gena in dorsal view approximately 0.7 times as long as width of eye; lower portion with longitudinal wrinkles, medially with dense punctures, upper portion smooth with very sparse and fine punctures. Vertex (Figure 4) smooth and shining, a few fine punctures. Interocellar area almost flat, with fine, dense punctures. Postocellar line about 1.7 times as long as ocular-ocellar line. Frons almost flat, with fine, dense, uneven punctures; median portion with fine longitudinal groove; lower portion deeply concave. Antenna relatively short, with 23 flagellomeres, each flagellomere longer than its diameter; penultimate flagellomeres 4 to 6 strongly curved, each flagellomere at curve with 2 peg-like setae. Ratio of length from first to fifth flagellomeres: 2.9:3.5:3.7:3.9:3.9. Occipital carina complete.

**Mesosoma.** Anterior portion of pronotum with fine, dense punctures; lateral concavity smooth and shining, remaining portion with dense, coarse punctures; dorsal portion, neck, with three strong, forking longitudinal carinae (Figure 6). Epomia very strong, upper end reaching to upper margin of pronotum, projecting and turned inward to dorsal centre of neck. Mesoscutum with dense, fine punctures. Middle lobe of mesoscutum (Figure 7) and anterior portion of lateral lobes with dense, distinct punctures. Mesoscutum with longitudinal wrinkles postero-medially, postero-laterally smooth and shining. Notaulus shallow, reaching 0.6 to 0.7 × distance to posterior margin of mesoscutum. Scutoscutellar groove smooth with strong median longitudinal carina. Scutellum rough, with irregular wrinkles, medially convex, subapical-medially concave. Postscutellum semicircular ridge-shaped convexity, anteriorly deeply concave. Mesopleuron (Figure 8) shining, evenly convex, with fine dispersed punctures; ventro-posteriorly with distinct transverse groove; without speculum; mesopleural fovea consisting of short, shallow horizontal groove near mesopleural suture. Upper end of epicnemial carina reaching subalar prominence. Metapleuron rough, with irregular reticulate wrinkles. Submetapleural carina complete. Wings hyaline. Fore wing with vein 1cu-a distal to 1/M by 0.25 to 0.5 × length of 1cu-a. Vein 2rs-m almost disappeared, approximately 0.15 × distance between it and 2m-cu. Vein 2-Cu approximately as long as or slightly longer than 2cu-a. Hind wing vein 1-cu approximately as long as cu-a. Legs relatively slender. Fore and mid tibiae very thick, approximately columnar, subbasal part of ventral side angularly concave. Front side of fore tibia with short spines. Hind coxa elongate, medially distinctly expanded. Claws relatively small. Propodeum rough, completely areolated. Area basalis small, triangular, apical part of lateral longitudinal carinae combined. Area superomedia pentagonal, costula connecting approximately at its middle. Area externa with oblique longitudinal wrinkles. Area dentipara with irregular transverse wrinkles. Areas superomedia and posteroexterna with vague, irregular or reticulate wrinkles. Area petiolaris with distinct longitudinal wrinkles. Propodeal spiracle small, elongate.

**Metasoma**. First tergum 1.7 to 1.8 × as long as apical width, rough, with weak oblique median groove from median lateral margin extending backward to posterior median part; anterior to spiracle with transverse wrinkles; medially with irregular reticulate wrinkles; posteriorly with irregular longitudinal wrinkles. Median dorsal and dorsolateral carinae present from base to spiracle. Spiracle slightly convex, at anterior 0.35 of first tergum. Second tergum approximately 0.76 × as long as apical width; anteriorly with distinct punctures, posteriorly with irregular longitudinal wrinkles; with deep oblique groove cutting off basolateral corner. Third tergum 0.55 to 0.6 × as long as apical width, with weak punctures, posteriorly with weak and indistinct transverse wrinkles. Terga 4 to 6 very short, with fine leathery texture. Last tergum in dorsal view triangular, dorsally concave, with smooth median longitudinal groove. Ovipositor sheath approximately 2.5 × as long as hind tibia. Ovipositor relatively slender, apical portion depressed. Apical part of lower valve with 7 inclivous ridges, basal 4 or 5 distinct and strong; basal of the ridges with a roughened area, length of roughened area approximately as long as distance between basal ridge and end of lower valve.

**Color** (Figure 1). Black, except the following: anterior profile of scape and flagellomeres 10 to 13 white. Clypeus blackish brown, along ventral margin vaguely yellowish brown. Basal portion of mandible dark red. Posterior portion of malar space with small brown spot. Fore and mid coxae (except basal portions brownish black), apical spots of fore and mid femora, main portions of anterior and posterior profiles of fore and mid tibiae, hind coxa dorsoapically, hind margins of terga 3 to 7 except dorso-lateral sides, white. Fore and mid legs irregularly dark brown. Hind trochanter, femur basally, tibia medially, tarsomere 1 (to 2) blackish brown. Apex of hind femur, both apices of hind tibia irregularly brown. Hind tarsomeres (2) 3 and 4 brown to light brown. Apical margin of tergum 1 narrowly brown. Stigma dark brown. Veins brownish black.

**Male** (Figure 2). Body length 5.0 to 5.2 mm. Fore wing length 3.3 to 3.4 mm. Antenna length approximately 5.5 mm. Flagellum (Figure 5) slightly compressed, apex of each flagellomere swollen, lateral and ventral-lateral profiles with erect, long setae, setae approximately 3.5 × as long as width of flagellomere, curved apically. Stigma approximately 3.2 × as long as width. Antenna entirely black. Terga entirely black, or second tergum and apex of first tergum more or less blackish brown.

**Cocoon** (Figure 12). About 8 to 10 mm long, median width about 1.5 to 2.0 mm. yellowish grey.

#### Host.

*Pterolophia alternata* Gressitt, 1938.

#### Host plant.

*Robinia pseudoacacia* L.

This new species is similar to *Xorides asiasius* Sheng & Hilszczański, 2009, *Xorides cinnabarius* Sheng & Hilszczański, 2009 and *Xorides sapporensis* (Uchida, 1928), possessing subapical terga with white spots on apical part in females; flagellomeres with perpendicular hairs about as long as or longer than diameter of flagellomere, stigma short and wide, approximately or less 3x as long as wide, first tergum with oblique median groove running from median lateral margin extending backward to posterior median portion in male (*Xorides asiasius* unknown). It can be distinguished from them by the following key.

##### Key to the similar species to *Xorides benxicus*

**Table:** 

1	Female	2
–	Male	5
2	Median dorsal carinae of first tergum reaching to hind margin of first tergite	3
–	Median dorsal carinae of first tergum at most reaching to median portion of first tergum	4
3	Terga 2 and 3 rough, with dense, indistinct punctures. Mesopleuron, propodeum, femora and first tergum red. Scutellum with white spot	*Xorides cinnabarius* Sheng & Hilszczański
–	Apical portion of tergum 2 and entire tergum 3 transversely aciculate. Mesopleuron, propodeum, scutellum, femora and first tergum entirely black	*Xorides sapporensis* (Uchida)
4	Clypeus with fine transverse lines. Fore wing vein 1cu-a opposite 1/M. Ovipositor sheath approximately 1.8 times as long as hind tibia. Ovipositor evenly and weakly down-curved, apically straight. Inner orbit, a large median spot on gena and apical spot on scutellum white. Terga 1 to 3 red. (Male unknown)	*Xorides asiasius* Sheng & Hilszczański
–	Clypeus slightly shagreened. Fore wing vein 1cu-a distal to 1/M. Ovipositor sheath approximately 2.5 times as long as hind tibia. Ovipositor straight, apically abruptly down-curved. Orbits, scutellum and terga 1 to 3 entirely black, except hind margin of tergum 1 narrowly reddish	*Xorides benxicus* Sheng, sp. n.
5	Terga 3 to 5, at least 4, transversely aciculate. Hind coxa and femur, metapleuron, propodeum and first tergum black	6
–	All terga entirely coarsely sculptured. Hind coxa and femur, at least parts of metapleuron, propodeum and first tergum red	*Xorides cinnabarius* Sheng & Hilszczański
6	Antennal flagellum weakly compressed, apical portion of each flagellomere swollen and with erect, long setae, setae approximately 3.5 times as long as width of flagellomere	*Xorides benxicus* Sheng, sp. n.
–	Antennal flagellum regular, apical portion of each flagellomere not swollen, setae approximately as long as width of flagellomere	*Xorides sapporensis* (Uchida)

**Figures 10–12.**
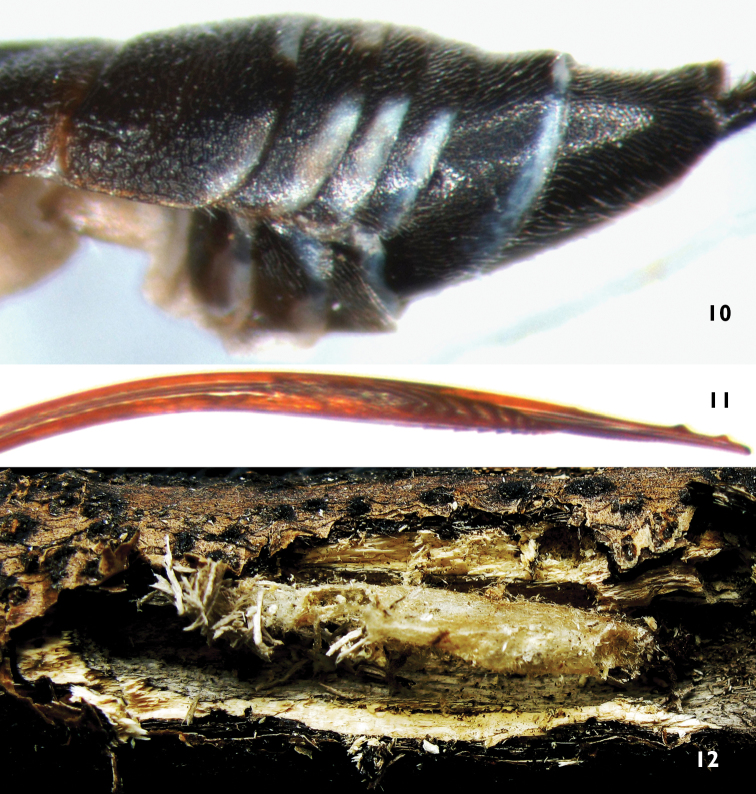
*Xorides benxicus* Sheng, sp. n. **10–11** Holotype female **10** Apical portion of metasoma, lateral view **11** Apical portion of ovipositor, lateral view **12** Cocoon.

## Supplementary Material

XML Treatment for Xorides

XML Treatment for Xorides benxicus

## References

[B1] Clément E (1938) Opuscula Hymenopterologica 4. Die paläarktischen Arten der Pimplinentribus Ischnocerini, Odontomerini, Neoxoridini und Xylomini (Xoridini Schm.). Festschrift für 60 Geburtst. Prof. Embrik Strand 4: 502-569

[B2] Gauld ID Wahl D Bradshaw K Hanson P Ward S (1997) The Ichneumonidae of Costa Rica, 2. Introduction and keys to species of the smaller subfamilies, Anomaloninae, Ctenopelmatinae, Diplazontinae, Lycorininae, Phrudinae, Tryphoninae (excluding Netelia) and Xoridinae, with an appendices on the Rhyssinae. Memoirs of the American Entomological Institute 57: 1-485

[B3] Hilszczański J Gibb H Hjältén J Atlegrim O Johansson T Pettersson RB Ball JP and Danell K (2005) Parasitoids (Hymenoptera, Ichneumonoidea) of Saproxylic beetles are affected by forest successional stage and dead wood characteristics in boreal spruce forest. Biological Conservation 126 (4): 456-464 doi: 10.1016/j.biocon.2005.06.026

[B4] Hilszczański J Plewa R (2011) Two new species of *Xorides* Latreille from Crete with a checklist of Greek Xoridinae Shuckard (Hymenoptera: Ichneumonidae). Annales Zoologici (Warsaw) 61 (3): 513-517

[B5] Liu T Sheng M-L (1998) Studyies on the genus *Xorides* (*Xorides*) from Northeast China. Entomologia Sinica 5 (1): 35-41

[B6] Mason WRM (1986) Standard drawing conventions and definitions for venational and other features of wings of Hymenoptera. Proceedings of the Entomological Society of Washington 88: 1-7

[B7] Mason WRM (1990) Cubitus posterior in Hymenoptera. Proceedings of the Entomological Society of Washington 92: 93-97

[B8] Meyer NF (1936) Parasitic Hymenoptera in the family Ichneumonidae of the USSR and adjacent countries. Part 5. Tryphoninae. Opredeliteli Faune SSSR 21 (5): 1-340

[B9] Ross HH (1936) The ancestry and wing venation of the Hymenoptera. Annals of the Entomological Society of America 29: 99-111

[B10] Sheng M-L (2002) A new species of genus *Xorides* from Henan Province (Hymenoptera: Ichneumonidae). In: Shen X-C (Ed.) The Fauna and Taxonomy of Insects in Henan, 5. Insects of the Mountains Taihang and Tongbai Regions. China Agricultural Scientech Press, Beijing, 42–44

[B11] Sheng M-L Hilszczański J (2009) Two new species of genus *Xorides* (Hymenoptera: Ichneumonidae) parasitizing *Saperda balsamifera* Motschulsky and *Asias halodendri* (Pallas) (Coleoptera: Cerambycidae) in China. Annales Zoologici (Warsaw) 59 (2): 165-170

[B12] Sheng M-L Huang W-Z (1999) Study on the genus *Xorides* from Funiu Mountains (Hymenoptera: Ichneumonidae). In: Shen X Pei H (Eds) The fauna and taxonomy of insects in Henan, 4. Insects of the mountains Funiu and Dabie Regions. China Agricultural Scientech Press, Beijing, 87–91

[B13] Sheng M-L Jiang S-Y (2006) A new species of Subgenus *Xorides* (Hymenoptera: Ichneumonidae: Xoridinae) from Oriental Part of China. Entomofauna 27: 189-192

[B14] Sheng M-L Kou M-J Cui Y-S Bing J-C Sun S-P Su W (2002) List of ichneumonids parasitizing wood boring insects in northern China. Journal of Gansu Forestry Science and Technology 27 (3): 1-5

[B15] Sheng M-L Lin X-A (2004) Subgenus *Moerophora* Förster of genus *Xorides* Latreille from North China (Hymenoptera: Ichneumonidae: Xoridinae). Linzer Biologische Beiträge 36 (2): 1055-1059

[B16] Sheng M-L Sun S-P (2009) Insect fauna of Henan, Hymenoptera: Ichneumonidae. Science Press, Beijing, China, 340 pp.

[B17] Sheng M-L Sun S-P (2010) Ichneumonids parasitizing wood-boring insect pests in China (Hymenoptera: Ichneumonidae). Science Press, Beijing, 380 pp.

[B18] Sheng M-L Wen J-B (2008) Species of subgenus *Xorides* Latreille (Hymenoptera, Ichneumonidae) parasitizing woodborers in palearctic part of China. Entomologica Fennica 19: 86-93

[B19] Sheng M-L Wu S-L (1998) Study on *Xorides* (*Moerophora*) (Hymenoptera: Ichneumpnidae) from northeastern China. Entomologia Sinica 5 (2): 113-116

[B20] Townes HK (1969) The genera of Ichneumonidae, Part 1. Memoirs of the American Entomological Institute 11: 1-300

[B21] Wahl DB (1997) The cladistics of the genera and subgenera of Xoridinae. Memoirs of the American Entomological Institute 57: 454–460 [in Gauld ID (Ed.) The Ichneumonidae of Costa Rica, 2]

[B22] Wang SF Gupta VK (1995) Studies on the Xoridine Ichneumonids of China (Hymenoptera: Ichneumonidae: Xoridinae). Oriental Insects 29: 1-21 doi: 10.1080/00305316.1995.10433737

[B23] Yu DS Horstmann K (1997) A catalogue of world Ichneumonidae (Hymenoptera). Memoirs of the American Entomological Institute 58: 1-1558

[B24] Yu DS van Achterberg C Horstmann K (2012) Taxapad 2012 - World Ichneumonoidea 2011. Taxonomy, Biology, Morphology and Distribution. On USB Flash drive. Ottawa, Ontario, Canada. http://www.taxapad.com

[B25] Zong S-X Sheng M-L (2009) A new species of genus *Xorides* Latreille (Hymenoptera. Ichneumonidae) from China. Acta Zootaxonomica Sinica 34 (4): 922-924

